# Supplementary material for: Towards a Rapid-Turnaround Low-Depth Unbiased Metagenomics Sequencing Workflow on the Illumina Platforms
Source: Bioengineering (Basel). 2023 Apr 25;10(5):520. doi: 10.3390/bioengineering10050520 (PMC10215873; doi:10.3390/bioengineering10050520)
Supplement: Supplementary file 1 [file bioengineering-10-00520-s001.zip › bioengineering-2344780-supplementary.pdf]

Supplementary Material

1 Supplementary Figures and Tables

1.1 Supplementary Figures

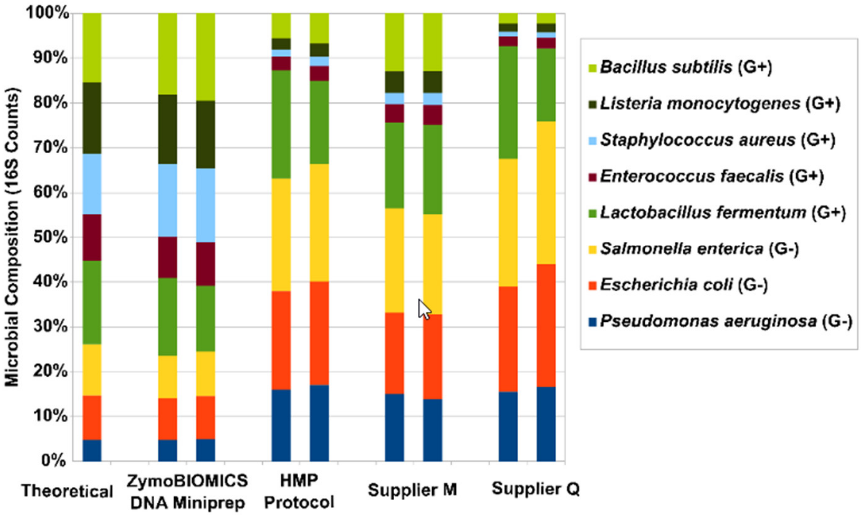

Supplementary Figure S1. Zymobiomics theoretical spike-in composition based on 16S rRNA amplicon sequencing (1).

1.2 Supplementary Tables

Species	Theoretical Composition (%)					Gram Stain
	Genomic DNA	16S Only	16S & 18S	Genome Copy	Cell Number	
Pseudomonas aeruginosa	12	4.2	3.6	6.1	6.1	-
Escherichia coli	12	10.1	8.9	8.5	8.5	-
Salmonella enterica	12	10.4	9.1	8.7	8.8	-
Lactobacillus fermentum	12	18.4	16.1	21.6	21.9	+
Enterococcus faecalis	12	9.9	8.7	14.6	14.6	+
Staphylococcus aureus	12	15.5	13.6	15.2	15.3	+
Listeria monocytogenes	12	14.1	12.4	13.9	13.9	+
Bacillus subtilis	12	17.4	15.3	10.3	10.3	+
Saccharomyces cerevisiae	2	NA	9.3	0.57	0.29	Yeast
Crytococcus neoformans	2	NA	3.3	0.37	0.18	Yeast

Supplementary Table S1. ZymoBIOMICS Microbial Community Standard (Zymo Research, Cat no. D6300) consist of 8 bacterial strains (3 Gram-negative and 5 Gram-positive) and 2 tough-to-lyse yeasts at a concentration of 1.4 x 10^{10} cells/mL (1).

Platform	Dilution Factor	No. of cells per mL of plasma	Categories	% of Reads (HostEL treated)	% of Reads (No treatment)	Fold Enrichment of Microbial reads
iSeq 100	1:100	~1.4 million	Homo sapiens	86.94%	93.18%	-
			Non Human	0.41%	0.23%	
			**Zymo Spike-ins**	**12.65%**	**6.61%**	**1.915**
	1:250	~560 000	Homo sapiens	90.70%	96.05%	-
			Non Human	0.37%	0.15%	
			**Zymo Spike-ins**	**8.94%**	**3.80%**	**2.355**
	1:1000	~140 000	Homo sapiens	96.36%	98.99%	-
			Non Human	0.24%	0.15%	
			**Zymo Spike-ins**	**3.40%**	**0.87%**	**3.932**
	1:2000	~70 000	Homo sapiens	98.46%	99.53%	-
			Non Human	0.21%	0.14%	
			**Zymo Spike-ins**	**1.32%**	**0.33%**	**3.952**
	No Spike	N.A.	Homo sapiens	99.87%	99.76%	-
			Non Human	0.08%	0.15%	
			**Zymo Spike-ins**	**0.04%**	**0.09%**	**0.407**
MiniSeq	1:100	~1.4 million	Homo sapiens	83.87%	91.79%	-
			Non Human	0.46%	0.28%	
			**Zymo Spike-ins**	**15.64%**	**7.91%**	**1.977**
	1:250	~560 000	Homo sapiens	87.76%	95.30%	-
			Non Human	0.46%	0.16%	
			**Zymo Spike-ins**	**11.75%**	**4.53%**	**2.594**
	1:1000	~140 000	Homo sapiens	93.33%	98.21%	-
			Non Human	2.42%	0.65%	
			**Zymo Spike-ins**	**4.24%**	**1.13%**	**3.752**
	1:2000	~70 000	Homo sapiens	97.50%	98.19%	-
			Non Human	0.44%	0.51%	
			**Zymo Spike-ins**	**2.05%**	**1.31%**	**1.565**
	No Spike	N.A.	Homo sapiens	99.50%	98.76%	-
			Non Human	0.15%	1.14%	
			**Zymo Spike-ins**	**0.34%**	**0.10%**	**3.4**

Supplementary Table S2. Detailed breakdown of percentage of reads of HostEL treated vs no treatment enrichment on iSeq 100 and MiniSeq sequencing platforms. HostEL can enrich the percentage of reads by ~2 fold in plasma with high microbial concentrations (1.4 million cells/mL) and ~4 fold with low microbial concentration (140,000 cells/mL and 70,000 cells/mL) on iSeq 100 sequencing platform while enriching at least 1.5 fold on MiniSeq sequencing platform.

Pathogen	Primer	Primer Sequence	Reference
BK virus	F	5′-AGT GTT GAG AAT CTG CTG TTG CTT-3′	(2)
	R	5′-GGG ATG AAG ATT TAT TTT GCC ATG AAG AT-3′	
Dengue Virus	F	5'-GCA TAT TGA CGC TGG GAR AGA C-3'	(3)
	R (DENV1-3)	5′-TTC TGT GCC TGG AAT GAT GCT G-3'	
	R (DENV4)	5′-YTC TGT GCC TGG ATW GAT GTT G-3′	
Hepatitis B virus	F	5'- TTC CGG AAA CTA CTG TTG TTA GAC-3'	(4)
	R	5'- ATT GAG ATT CCC GAG ATT GAG A-3'	
Hepatitis C virus	F	5'-GTC TGC GGA ATC GGT GAG TAC A-3'	(5)
	R	5'-CTA TCA GGC AGT ACC ACA AGG-3'	
Hepatitis E virus	F	5′-AAT AAA TCA TAA GGT GGT TTC TGG GGT GAC-3′	(6)
	R	5′-AAT AAA TCA TAA GGG GTT GGT TGG ATG AA-3′	
Varicella-Zoster Virus	F	5'-CAA AGC GGG TCC ATC CCT-3′	(7)
	R	5'-ACC TGG CCT TTG CCG G-3′	

Supplementary Table S3. List of targeted primers used in qPCR validation of clinical samples.

Samples	qPCR Ct Value	Frac Reads	Raw Reads Counts	Platform	Clinical Diagnosis
B047	43.8	0.00E+00	0	iSeq 100	Hepatitis B virus
B046	UD	0.00E+00	0		
B050	UD	0.00E+00	0		
B048	38.6	8.00E-05	2		
B049	29.5	8.05E-03	118		
B017	24.4	2.11E-02	495		
B016	17.9	8.64E-02	4238		
B045	22.1	3.32E-01	7427		
B047	43.8	0.00E+00	0	MiniSeq	
B046	UD	0.00E+00	0		
B050	UD	0.00E+00	0		
B048	38.6	2.00E-05	2		
B049	29.5	7.80E-03	318		
B017	24.4	0.02203	1226		
B016	17.9	0.09309	11394		
B045	22.1	3.77E-01	23336		

Supplementary Table S4. qPCR and iSeq 100/MiniSeq sequencing results of Hepatitis B plasma samples

2 References

1. ZymoBIOMICS^{TM} Microbial Community Standard (D6300) [Internet]. [cited 2022 Mar 29]. Available from: https://files.zymoresearch.com/protocols/_d6300_zymobiomics_microbial_community_standard.pdf

2. Pinto GG, Poloni JAT, Paskulin DDA, Spuldaro F, Paris F de, Barth AL, et al. Quantitative detection of BK virus in kidney transplant recipients: a prospective validation study. Jornal Brasileiro de Nefrologia [Internet]. 2018 Jan 1 [cited 2022 May 11];40(1):59. Available from: /pmc/articles/PMC6533964/

3. Alm E, Lesko B, Lindegren G, Ahlm C, Söderholm S, Falk KI, et al. Universal Single-Probe RT-PCR Assay for Diagnosis of Dengue Virus Infections. PLoS Negl Trop Dis [Internet]. 2014 Dec 18 [cited 2022 May 11];8(12). Available from: /pmc/articles/PMC4270494/

4. Liu C, Chang L, Jia T, Guo F, Zhang L, Ji H, et al. Real-time PCR assays for hepatitis B virus DNA quantification may require two different targets. Virol J [Internet]. 2017 May 12 [cited 2022 May 11];14(1). Available from: /pmc/articles/PMC5427580/

5. Warkad SD, Nimse SB, Song KS, Kim T. Development of a Method for Screening and Genotyping of HCV 1a, 1b, 2, 3, 4, and 6 Genotypes. ACS Omega [Internet]. 2020 May 19 [cited 2022 May 11];5(19):10794–9. Available from: https://pubs.acs.org/doi/full/10.1021/acsomega.0c00386

6. Germer JJ, Ankoudinova I, Belousov YS, Mahoney W, Dong C, Meng J, et al. Hepatitis e virus (HEV) detection and quantification by a real-time reverse transcription-PCR assay calibrated to the world health organization standard for HEV RNA. J Clin Microbiol [Internet]. 2017 May 1 [cited 2022 Oct 7];55(5):1478–87. Available from: https://journals.asm.org/doi/10.1128/JCM.02334-16

7. Harbecke R, Oxman MN, Arnold BA, Ip C, Johnson GR, Levin MJ, et al. A Real-Time PCR Assay to Identify and Discriminate Among Wild-Type and Vaccine Strains of Varicella-Zoster Virus and Herpes Simplex Virus in Clinical Specimens, and Comparison With the Clinical Diagnoses. J Med Virol. 2009;81(7):1310–22.
